# Multiple-Localization and Hub Proteins

**DOI:** 10.1371/journal.pone.0156455

**Published:** 2016-06-10

**Authors:** Motonori Ota, Hideki Gonja, Ryotaro Koike, Satoshi Fukuchi

**Affiliations:** 1 Graduate School of Information Sciences, Nagoya University, Nagoya, Japan; 2 Faculty of Engineering, Maebashi Institute of Technology, Maebashi, Japan; Youngstown State University, UNITED STATES

## Abstract

Protein-protein interactions are fundamental for all biological phenomena, and protein-protein interaction networks provide a global view of the interactions. The hub proteins, with many interaction partners, play vital roles in the networks. We investigated the subcellular localizations of proteins in the human network, and found that the ones localized in multiple subcellular compartments, especially the nucleus/cytoplasm proteins (NCP), the cytoplasm/cell membrane proteins (CMP), and the nucleus/cytoplasm/cell membrane proteins (NCMP), tend to be hubs. Examinations of keywords suggested that among NCP, those related to post-translational modifications and transcription functions are the major contributors to the large number of interactions. These types of proteins are characterized by a multi-domain architecture and intrinsic disorder. A survey of the typical hub proteins with prominent numbers of interaction partners in the type revealed that most are either transcription factors or co-regulators involved in signaling pathways. They translocate from the cytoplasm to the nucleus, triggered by the phosphorylation and/or ubiquitination of intrinsically disordered regions. Among CMP and NCMP, the contributors to the numerous interactions are related to either kinase or ubiquitin ligase activity. Many of them reside on the cytoplasmic side of the cell membrane, and act as the upstream regulators of signaling pathways. Overall, these hub proteins function to transfer external signals to the nucleus, through the cell membrane and the cytoplasm. Our analysis suggests that multiple-localization is a crucial concept to characterize groups of hub proteins and their biological functions in cellular information processing.

## Introduction

Eukaryotic cells are composed of many subcellular compartments, and each provides a specific environment for proteins to function [1]. For instance, each cell has a nucleus, in which a set of chromosomes is stored and genetic information is processed. Transcription factors, activators, repressors, and mediators cooperate with other related factors to elegantly regulate transcription, and polymerases synthesize DNA and RNA. In the cytoplasm, many metabolic reactions are conducted by a variety of enzymes. They are engaged in catabolism and anabolism, using ATP supplied by mitochondria. Membranes surround the cell and separate it from the outside environment. All of the materials required by a cell are imported through the cell membranes by transporter or pump proteins. Receptors receive various signals from the environment outside the cell, and transmit them to the inside. These examples indicate the strong relationships between the subcellular localization of a protein and its function. Thus, the subcellular localization provides a significant clue for the identification of protein function [2, 3]. Numerous experimental [4, 5] and computational methods [6, 7] have been developed to determine and to infer the subcellular localizations of proteins.

In the subcellular compartments, most proteins interact with other proteins for their functions. In this sense, protein-protein interactions (PPIs) are fundamental to support biological phenomena. During the past decade, high-throughput and proteome-wide methods to investigate PPIs have been applied, to obtain PPI data for many eukaryotic organisms [8–11]. These interaction data are represented by network graphs and analyzed by network science methods [12]. The PPI network is scale-free [11, 13, 14]; that is, the distribution of the number of interactions for each protein follows the power law. In such a network, a small number of proteins interact with numerous proteins, while most of the others interact with only a few proteins. The proteins with numerous interaction partners are called hub proteins. Hub proteins are attracting keen attention [15–18], because they are usually situated at the center of the network, and connect many network modules [19]. As a result, the hub proteins are likely to be essential proteins for the organisms; i.e., their knock-out results in lethality [13].

Various attributes that distinguish the hub proteins from the non-hub proteins have been reported. The hub proteins tend to be composed of many repetitive or distinct structural domains [15], together with substantial intrinsically disordered regions (IDRs) [16]. The biological processes in which they function tend to be transcription and signal transduction [14, 20, 21], and they undergo multiple post-translational modifications (mPTM). However, to date, the relationships between the hub proteins and the subcellular localizations have not been explicitly described. It is intriguing that intrinsically disordered proteins (IDPs) are abundantly localized in the nucleus [22, 23], and the hub proteins have a significant number of IDRs. Does this imply that the hub proteins are frequently found in the nucleus? This question has not been answered yet.

**Fig 1:**
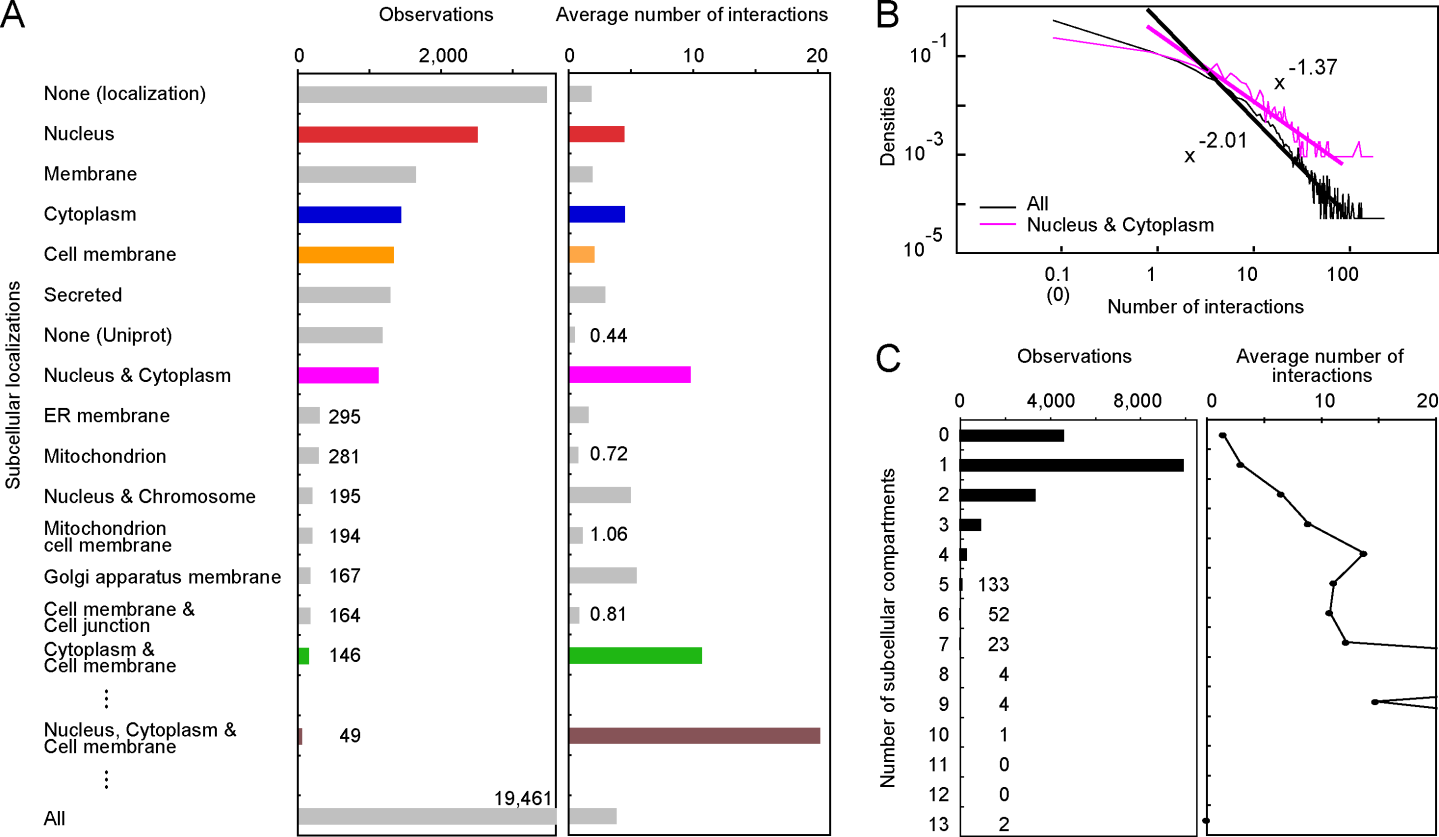
The statistics of the subcellular localizations and the numbers of interactions. A. The number of proteins (observations) in each subcellular localization and the average number of interactions are indicated, in the descending order of the observations. The data for NP, CP, MP, NCP, CMP, and NCMP are colored red, blue, orange, magenta, green and brown, respectively. None (locatization) means that no subcellular localization was denoted in Uniprot, and None (Uniprot) means that no Uniprot entry was assigned to the HPRD entry. The observations represented by the short bars are shown as numbers, and the number by the bottom bar is the total number of HPRD entries. B. The distribution of the number of interactions in the log-log plot. When the number of interactions was zero, the data are shown at 0.1 interactions. The distribution was approximated by the power function, using the data from 1 to 100 interactions. The scaling exponents are -2.01 and -1.37 for the distributions of all proteins and the NCP, respectively. C. The statistics of the numbers of subcellular compartments for each HPRD entry, and the average number of interactions against the numbers of subcellular compartments. The full size view of the right panel is shown in S1 Fig.

In this study, we re-investigated the numbers of interactions of human proteins, in terms of their subcellular localizations, based on the Human Protein Reference Database (HPRD) [24] and Uniprot [25]. In most of the previous studies, each of the subcellular localizations was evaluated in a one-by-one manner, in which the number of interactions was examined for each subcellular compartment. This approach is effective if almost every protein is localized only in a single subcellular compartment, but it cannot address the issue of multiple localizations [26]. However, a quarter of the proteins in HPRD are localized in multiple subcellular compartments, according to Uniprot (see Fig 1C shown below). In such a case, the one-by-one manner may multiply count the number of interactions for each of multiple subcellular compartments [14], or may entirely discard them [3]. Instead of adopting the one-by-one approach, we prepared the categories of subcellular localizations for the proteins that can be localized in multiple subcellular compartments, or translocated from one subcellular compartment to another. The recent report by Huang et al. [27] also considered multiple localizations. However, they mainly discussed the hub proteins from the viewpoints of mPTM and diseases. In this report, we directly analyze the relationships between multiple localizations and hub proteins, and also highlight their features, through surveys of the keyword annotations, domain architectures, and intrinsic disorder.

## Results and Discussion

### Subcellular localization and number of interactions

The binary PPI data of the human proteome were manually curated and deposited in HPRD [24]. Referring to the Uniprot accession, we assigned each of the HPRD proteins to the Uniprot entry [25] (as of Feb. 2015), in which the subcellular localization was described while allowing multiple subcellular compartments. The statistics of the subcellular localizations are shown in the descending order of the number of proteins at each localization (Fig 1A). For 6 localizations (nucleus, membrane, cytoplasm, cell membrane, secreted, and both nucleus and cytoplasm), the numbers of observations are more than 1,000, but there is a significant gap to the next major group: the number of proteins localized in the ER membrane is less than 300. The average number of interactions was calculated for the proteins in each subcellular localization (Fig 1A, right panel). The proteins solely localized in either the nucleus (the nucleus proteins: NP, hereafter) or the cytoplasm (the cytoplasm proteins: CP), have about 4.5 interaction partner proteins (4.41 and 4.45, respectively), and their values are larger than those of the proteins in the other single subcellular compartments, with more than 100 observations [3] (the third largest is 2.83 interactions for secreted proteins). Notably, the proteins localized in both the nucleus and cytoplasm (the nucleus/cytoplasm proteins: NCP) have 9.75 interaction partners on average, and thus twice as many interactions as NP or CP. The proteins localized in the cytoplasm/cell membrane (CMP) have 10.67 interactions, although they are only observed 146 times. These values are prominent among the proteins in each localization with more than 100 observations. Among the proteins observed more than 30 times, the tri-localized proteins in the nucleus/cytoplasm/cell membrane (NCMP) have the most interaction partners (20.10 interactions, 49 observations). These results indicate that multiple-localization is likely to be a significant characteristic of the hub proteins. All of the statistics are shown in S1 Table.

The distribution of the numbers of interactions for the aforementioned proteins, such as NCP (magenta in Fig 1B), also follows the power law (the coefficient of determination (R^2^) = 0.896), and is qualitatively the same as the previous results [11, 13, 14] and for all proteins in HPRD (black, R^2^ = 0.922). However, the absolute value of the scaling exponent is smaller (-1.37), meaning that the frequency of non-hub proteins in NCP is smaller than that of all proteins, and the frequency of hub proteins is larger than that of all proteins. Note that not all NCP (CMP or NCMP) are hub proteins. This is similar to the case for the other previously mentioned characteristics of hub proteins; for example, not all multi-domain proteins are hubs, but these proteins show a strong tendency to be hub proteins, as compared with the single-domain proteins [15].

It is naturally assumed that a given protein only interacts with another one if they can meet somewhere. In other words, two interacting proteins should co-exist within the same subcellular compartment. When a protein is only localized within a single subcellular compartment, the number of interactions is limited. Consequently, multiple-localization is reasonable, as a characteristic of hub proteins. In HPRD, the subcellular localizations of one quarter of the proteins (4,632 among 19,461) are unknown (the bin labeled “0” in the left panel of Fig 1C). Two-thirds of the rest (half of the total proteins, 9,955) are proteins localized only in one subcellular compartment, and one-third (one-quarter of the total, 4,874) are proteins localized in multiple subcellular compartments. Although the single subcellular localization is common, the occurrence of multiple subcellular localizations is not negligible [3]. For the multiple-localized proteins, we noticed that the average number of interactions tends to increase as the number of subcellular compartments increases (right panel of Fig 1C). Then, we statistically tested if an increment in the number of subcellular compartments was effective to increase the number of interactions. As a result, for 2, 3, and 4 subcellular compartments, an increment in the number of subcellular compartments significantly contributed to an increase in the numbers of interactions (S2 Table). We further examined whether the localization of specific multiple-subcellular compartments is statistically important for the large number of interactions. For example, we compared the distribution of the number of interactions in NCP with that of all bi-localized proteins (S3 Table). As a consequence, NCP and CMP are the only bi-localized proteins with statistics of interactions that are significantly biased to be large, as compared with those of all bi-localized proteins. The same procedure was applied for the tri- and quad-localized proteins, and only NCMP was selected to be hub proteins (S4 and S5 Tables). Thus, NCP, CMP and NCMP are the multiple-localized hub proteins, in which their specific subcellular localizations, in addition to the number of subcellular compartments, are important for their interactions with numerous proteins.

### Interaction partners of the multiple-localized hub proteins

**Fig 2:**
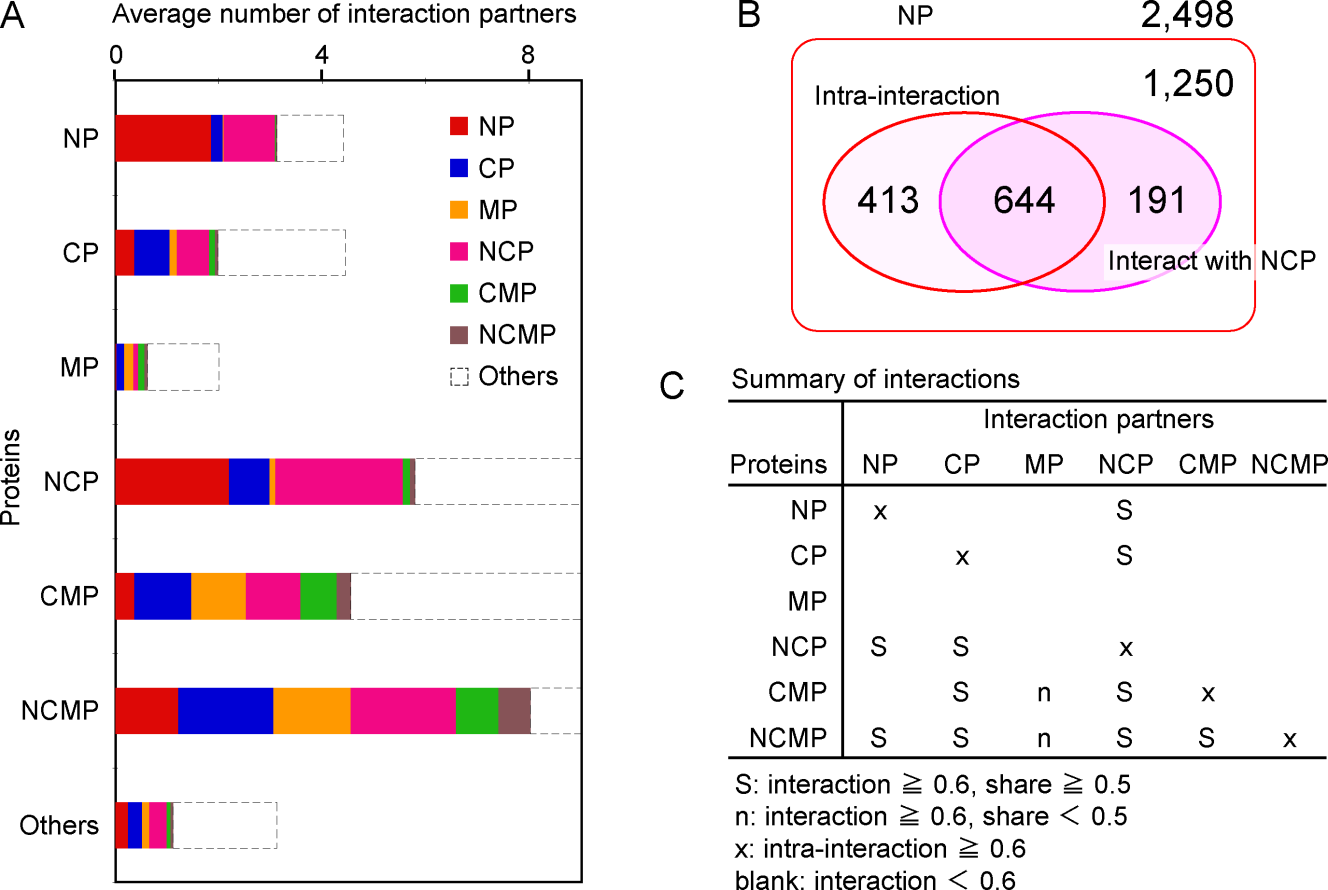
A. The average number of interactions decomposed by the interaction partners in 7 categories: NP, CP, MP, NCP, CMP, NCMP, and others. The full size view is shown in S2 Fig. B. The Venn diagram representing the interaction of NP with NCP. In total, 2,498 NP were analyzed. The NP with intra-interactions and interacting with NCP are shown by the two circles in the box. The intersection of two circles represents the shared interaction partners. Among these proteins, 1,250 NP neither intra-interact nor interact with NCP. C. The summary of interactions in terms of interaction partners. S symbols indicate that the interaction is rich (at least 0.6 interaction partners) between the protein (the left column) and the interaction partners (the top row), and the interaction partners are shared (at least 0.5) with the intra-interactions of partner proteins (S6 Table). For other symbols, see the bottom of the panel.

To characterize the interactions of NCP, CMP and NCMP, we examined the subcellular localizations of the interaction partners. We divided all of the proteins into 7 categories: NP, CP, MP (cell membrane proteins), NCP, CMP, NCMP, and others, and decomposed the average numbers of interactions (bars in the right panel of Fig 1A) into 7 groups, according to the interaction partners (Fig 2A). A high degree of decomposed interactions indicates that the interactions are enriched between the groups of proteins. In general, intra-interactions (interactions among the same groups) are abundant. When the decomposed interaction is defined as being rich if it is greater than 0.6, all intra-interactions are rich, except those of MP. Thus, we adopted 0.6 as the threshold of a rich interaction. For example, in NP (the top bar in Fig 2A), the intra-interactions, and the interactions with NCP are rich. In NCP, the interactions with NP and CP, and the intra-interactions are rich. Considering the similar number of the decomposed interactions between NCP and NP (the red part of the NCP bar), and that of the intra-interactions among NP (the red part of the NP bar), we suspect that the interaction partners in NP are common for NCP and NP. Among the 2,498 NP (Fig 2B), 1,057 (644+413) have at least one intra-interaction, and 835 (644+191) have at least one interaction with NCP. The intersection of both comprises 644 NP. That is, 644 NP can interact with NP as well as NCP. Conversely, this indicates that they are the common interaction partners shared by NP and NCP. Among NP interacting with NCP (835), more than 70% (644) have intra-interaction partners of NP. Among A interacting with B, when more than half of them also have intra-interaction partners of A, we thus consider the interaction partner to be “shared”. S6 Table presents the share rates of the interaction partners between proteins in different groups. In Fig 2C, the interactions are summarized in terms of the interaction partners. Except for the interaction with MP, the multiple-localized hub proteins interact well (richly) with the local proteins that are only localized in a single subcellular compartment, and the interaction partners are shared with the local proteins. In addition, they interact well with the other group of multiple-localized hub proteins, if they could co-exist in at least one subcellular compartment. This kind of promiscuity is one of the reasons why these multiple-localized proteins can interact with numerous proteins.

### Functions and features of the multiple-localized hub proteins

We assumed that some molecular functions or features were the specific factors contributing to the large number of interactions by NCP, CMP and NCMP. To validate this hypothesis, we evaluated the enrichments of their Uniprot keywords, and compared them with those of NP, CP and MP (S7 Table). In NCP, the keywords related to post-translational modifications (PTM), such as phosphoprotein, acetylation, and Ubl conjugation, are significantly overrepresented (see the Z-score derivation in S1 Document). The enrichment of keywords related to transcription is evident for NP. The keywords of transcription, activator and repressor are also frequent, but to a lesser extent, in NCP. Acetylation is abundant in both NCP and CP, but no other keyword is commonly overrepresented. In CMP and NCMP, kinase-related keywords (kinase, nucleotide-binding, ATP-binding, phosphoprotein) are evident. Apparently, the enrichments of their keywords are different from those of MP, indicating that CMP and NCMP are not typical membrane proteins.

**Fig 3:**
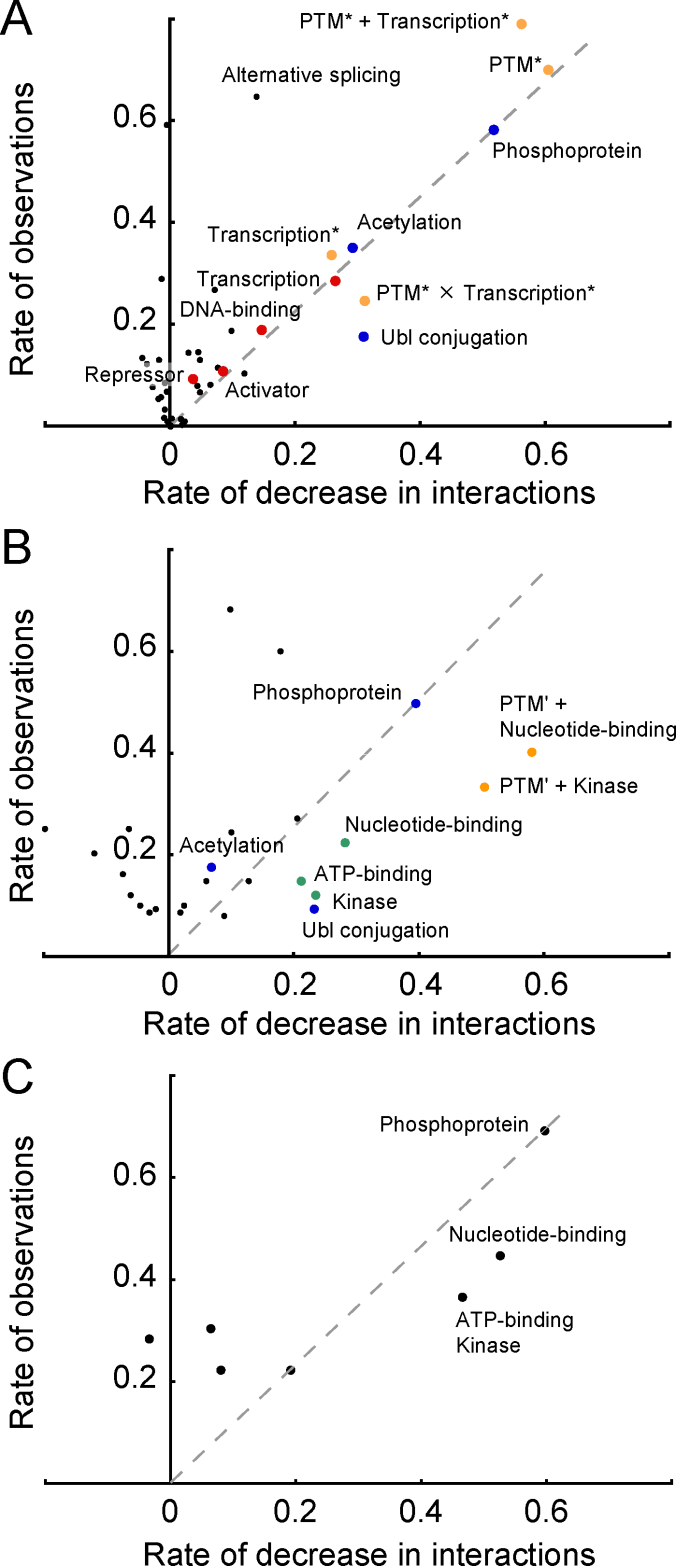
Scatter plot of the decrease in the average number of interactions after eliminating the proteins with the corresponding keyword, and the number of eliminated entries. The horizontal and the vertical data are the rates normalized by the original average number of interactions (for NCP: 9.75) and the total number of entries (for NCP: 1,120). A. NCP. The keywords related to post-translational modifications and transcription are shown by blue and red dots, respectively. PTM* is the union of the “phosphoprotein”, “acetylation” and “Ubl conjugation” keywords. Transcription* is the union of the “transcription”, “DNA-binding”, “activator” and “repressor” keywords. PTM* + Transcription* and PTM* × Transcription* are the union and the intersection of PTM* and Transcription*, respectively. These groups of unified keywords are shown in orange. Keywords that appeared more than 50 times were examined. B. CMP. The keywords related to the kinase activity are next to the green dots. PTM’ is the union of the “Ubl conjugation” and “acetylation” keywords. PTM’ + Kinase and PTM’ + Nucleotide-binding are the unions of PTM’ and respective keywords, shown with orange dots. C. NCMP. In B and C, keywords that appeared more than 10 times were examined.

Although the analysis of the keyword-enrichment is helpful for the functional characterization, it is unclear if the enriched features (keywords) actually contribute to the large number of interactions. To assess the contribution of each keyword, we re-calculated the average number of interactions by eliminating the proteins annotated by a specific keyword. When the decrease in the average number of interactions from the original figure (for NCP: 9.75) is large, the contribution of the eliminated proteins to the number of interactions is remarkable. We also noticed that the contribution generally depended on the number of proteins with a keyword. Thus, we plotted the observations of the proteins eliminated from the original statistics against the decrease in the average number of interactions (Fig 3).

In NCP (Fig 3A), for the keywords of phosphoprotein, Ubl conjugation, acetylation, transcription, and DNA-binding, the decrease rate is more than 0.1, reflecting their considerable contributions to the number of interactions. Although almost all of the points are plotted above the dashed gray line (the implications of the line are discussed in S1 Document), the point of Ubl conjugation is located below the line. This indicates that the contribution of proteins with the keyword is effective, considering its observations. We disregarded the alternative splicing keyword even though the decrease rate is also more than 0.1, because the number of observations is large (727). Considering these analyses collectively, NCP, as hub proteins, are roughly characterized by PTM and transcription. The two groups of keywords are hereafter denoted as PTM* (the union of the phosphoprotein, Ubl conjugation and acetylation keywords) and transcription* (the union of the transcription, DNA-binding, activator and repressor keywords). Actually, these features are responsible for numerous interactions (blue and red dots in Fig 3). The contributions of PTM*, transcription*, and the union and the intersection of both groups (PTM* + transcription* and PTM* × transcription*, respectively) were evaluated (Fig 3A, orange dots). Only the intersection of PTM* and transcription* (278 relevant proteins with an average number of 18.9 interactions) shows a contributive decrease (the decrease rate is 0.31) and is effective (below the dashed gray line). Consequently, we conclude that both PTM and transcription are likely to be important factors to identify the functions and features of NCP as the hubs. It is easily anticipated that the states of proteins alter with PTM. The alteration of the state frequently induces the association or dissociation with the interaction partners, resulting in more interactions. It is also plausible that PTM leads to the translocation from the cytoplasm to the nucleus and *vice versa*, directly or indirectly. For instance, in β-catenin [28, 29] and p53 [30], phosphorylation and ubiquitination control the protein concentration to initiate the transfer to the nucleus. In the nucleus, the proteins function as transcription factors or regulators interacting with various NP. This scenario presents a hypothetical explanation for the relationships between PTM and transcription with the large number of NCP interactions. We will verify this idea later with some examples.

We conducted the same analysis for CMP (Fig 3B). As a result, we conclude that the proteins annotated by the Ubl conjugation, acetylation or nucleotide-binding keywords (PTM’ + Nucleotide-binding) are the most significant and effective for the large number of interactions (the orange dots in Fig 3B). Note that the group of proteins annotated with nucleotide-binding (33 proteins) includes groups with ATP-binding or kinase. Due to the small number of observations, we plotted the data of a single keyword for NCMP, and did not perform the further analysis (Fig 3C). Proteins with kinase-related keywords are significant and effective in NCMP.

### Structural features of the multiple-localized hub proteins

**Fig 4:**
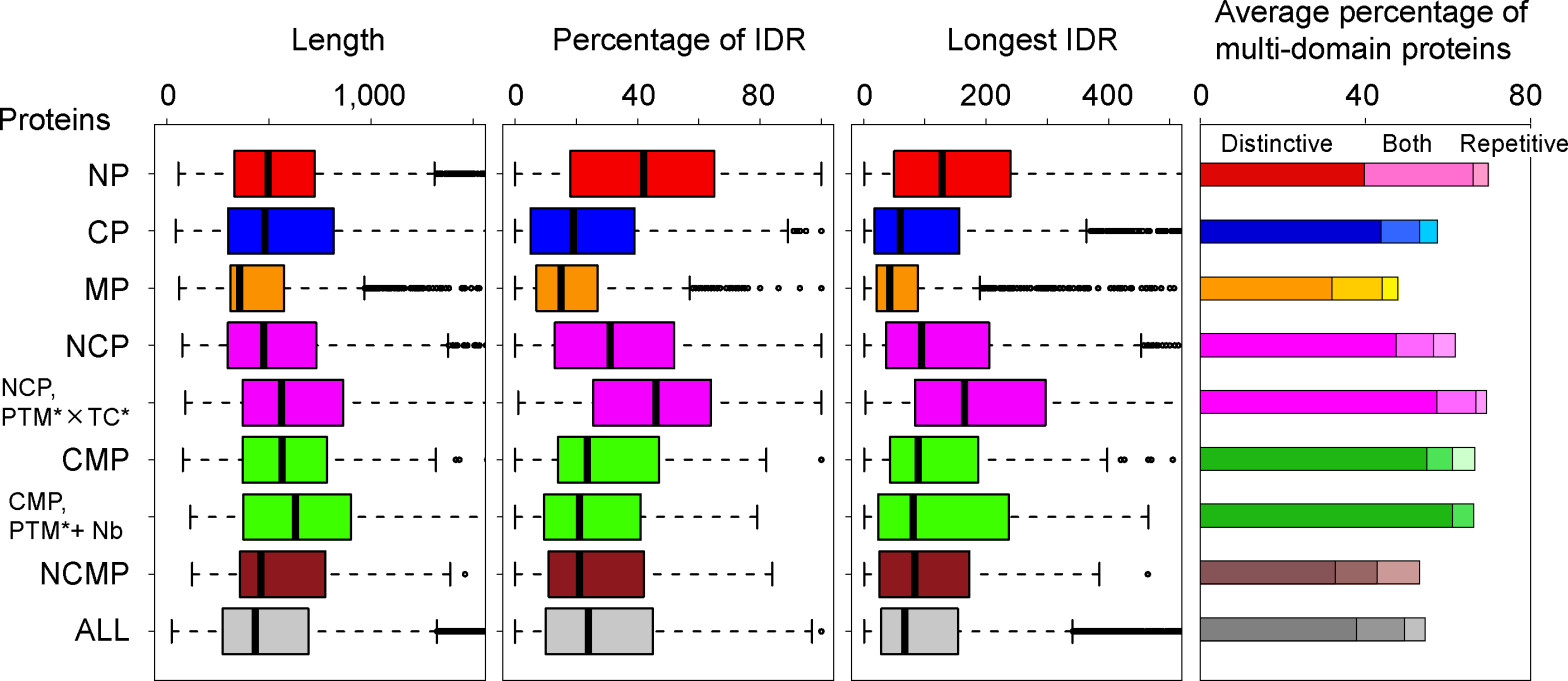
The protein structures characterized according to the intrinsic disorder and the domain architecture. From the left, the distributions of protein length, percentage of IDR, and longest IDR length are shown (see full size view in S3 Fig). The average percentage of multi-domain proteins is presented in the right panel, where the proteins were divided into those composed of only distinctive multi-domains (D), distinctive and repetitive multi-domains (B), and only repetitive multi-domains (R). The compositions are represented by the brightness of the colors. TC* and Nb are the abbreviations of transcription* and nucleotide-binding.

We analyzed the structures of NCP, CMP and NCMP, in terms of their intrinsic disorder and domain architectures. The results were compared with those from NP, CP and MP. The IDRs were predicted by DICHOT [31]. The P-fam domains [32] were assigned by HMMER [33]. The distributions of the protein length, the percentage of IDR, and the longest IDR length are shown in Fig 4, along with the average percentage of multi-domain proteins (see S8 Table for statistical significance). First, we focused on NCP. The IDRs and the multi-domain proteins are abundant in NP (red), and scarce in CP (blue). In NCP (upper magenta bars), the percentages of IDRs and multi-domain proteins are intermediate between NP and CP, and slightly more frequent than that of all proteins (gray). Qualitatively, the same results were obtained using Disopred2 [22] as the predictor of IDR. The protein lengths are almost the same among NP, CP, and NCP (the p-values of the Mann-Whitney U test are more than 0.1), but longer than that of all proteins (S8 Table). The same analysis was applied to NCP annotated by the PTM* × transcription* keywords (lower magenta bars). The proteins in this group are long. They include substantial IDRs and tend to be multi-domain proteins, comparable to NP, probably reflecting the fact that the PTM target residues are frequently found in IDRs [34]. It has been pointed out that an abundance of IDRs and multi-domain proteins is characteristic of the hub proteins [14–16, 20, 21]. In this sense, these proteins exhibit the typical features of the hub proteins. Although the average percentages of multi-domain proteins are similar in NP (69.7%) and in NCP with the PTM* × transcription* keywords (69.2%), we noticed that the contents were different: The latter indicates the strong preference toward multi-distinctive domains, instead of multi-repetitive domains (S1 Document). The intrinsic disorder is not evident in CMP and NCMP. For CMP, the average percentage of multi-domain protein is high, with a preference for multi-distinctive domains. When we applied TMHMM [35] for the prediction of transmembrane helices, we noticed that CMP and NCMP were different from MP. For MP, CMP, and NCMP, the lengths of the transmembrane regions are respectively 106.4 (20.5%), 12.7 (1.9%), and 3.2 (0.5%), on average. This indicates that most of CMP and NCMP do not include substantial transmembrane regions. Considering their multiple-localizations, they would be closely associated with, or attached to the cytoplasmic side of the membrane. Actually, about one-third of CMP and NCMP are annotated as peripheral membrane proteins in Uniprot, while the rate is only less than 2% in MP.

It is considered that the relationship between multiple-localizations and hub proteins as well as their structural features would be promising information in various research areas. For instance, it is helpful to improve the prediction of subcellular localization of proteins [6, 7], or to identify hypothetical hub proteins [36].

### Typical examples of the multiple-localized hub proteins

#### NCP related to post-translational modifications and transcription

Table 1 contains a list of the hub proteins interacting with more than 100 partner proteins (the PPI column), localized in both the nucleus and cytoplasm, and marked by the Uniprot keywords (the keyword column) of PTM* × transcription* (see also S9 Table: the complete list of hub proteins in NCP). We surveyed their functions and briefly summarized them in the function column. Notably, most of them are transcription factors. Except for mitogen-activated protein kinase 1, the proteins are principally involved in transcription. The biological processes in which they participate are shown in the process column. Most of them are involved in signaling pathways, in which the cell receives a signal from the extracellular environment and responds to it. To react to the signal, the expression of several genes is required, and the transcription occurs in the nucleus. Therefore, the reaction process must transfer the external cell signal into the nucleus via the cytoplasm. This type of transcription factor is known to translocate from the cytoplasm to the nucleus in the signaling pathways, and phosphorylation triggers the translocation (P in the translocate column). Ubiquitin-associated proteolysis is also known to regulate these signaling pathways.

**Table 1:** Hub proteins localized in both the nucleus and cytoplasm, annotated by PTM* and transcription* (more than 100 PPIs)

protein	Uniprot	HPRD	PPI	keyword	length	%ID	LID	domains	function	process	TL	IDEAL	ProS
Histone acetyltransferase p300	Q09472	4078	209	P/Ac/U/T	2414	57	319	8	TcoA	TC		70	
CREB-binding protein (CBP)	Q92793	2534	198	P/Ac/U/T/Av	2442	61	340	8	TcoA	TC		92	x
Mothers against decapentaplegic homolog 3 (Smad3)	P84022	4380	182	P/Ac/U/T/D	425	16	57	2	TF	Smad	P	113	x
Mothers against decapentaplegic homolog 2 (Smad2)	Q15796	3221	165	P/Ac/U/T/D	467	14	60	2	TF	Smad	P	127	x
Mitogen-activated protein kinase 1 (MAPK1)	P28482	1496	160	P/Ac/U/T/D/R	360	4	11	1	PK	MAPK	P		
Ataxin-1	P54253	3333	159	P/U/T/D/R	815	42	118	3	CB	Notch			
Mothers against decapentaplegic homolog 4 (Smad4)	Q13485	2995	150	P/Ac/U/T/D	552	31	164	2	TF	Smad	P	132	
Androgen receptor (AR)	P10275	2437	150	P/U/T/D/Av	919	60	554	4	NR/TF	NR	L	20	
Coiled-coil domain-containing protein 85B (Ccdc85B)	Q15834	16101	129	Ac/T/R	202	40	51	1	TcoR				
Transcription factor p65	Q04206	1241	113	P/Ac/U/T/D/Av	551	45	231	1	TF	NF-κΒ	P	207	x
Mothers against decapentaplegic homolog 9 (Smad9)	O15198	4484	110	P/T/D	467	24	102	2	TF	Smad	P		
Mothers against decapentaplegic homolog 1 (Smad1)	Q15797	3356	109	P/Ac/U/T/D	465	23	103	2	TF	Smad	P	174	x
Signal transducer and activator of transcription 3 (STAT3)	P40763	26	101	P/Ac/T/D/Av	770	9	55	4	TF	JAK/STAT	P		

Abbreviations: Uniprot, Uniprot accession; HPRD, HPRD ID; PPI, number of PPIs; in the keyword column, P, phosphoprotein; Ac, acetylation; U, Ubl conjugation; T, transcription; D, DNA-binding; Av, activator: R, repressor; %ID, percentage of IDR; LID, length of the longest IDR; domain, number of domains; in the function column, TcoA, transcription co-activator; TF, transcription factor; PK, protein kinase; CB, chromatin binding; NR, nuclear receptor; TcoR, transcription co-repressor; in the process section, TC, transcription; Smad, Smad signaling pathway; MAPK, MAPK cascade; Notch, Notch signaling pathway; NR, nuclear receptor signaling pathway; NF-κΒ, NF-κΒ signaling pathway; JAK/STAT, JAK/STAT signaling pathway; TL, trigger of translocation into nucleus; P, phosphorylation; L, ligand binding; IDEAL, IDEAL identifier; in the ProS column, x, existence of protean segments.

In the annotation process of each protein, we referred to IDEAL, a database of IDPs developed by our group [37, 38]. For some of the IDPs in Table 1, IDEAL provides experimental evidence for their unique features (IDEAL identifiers are shown in the IDEAL column). Especially, IDEAL compiles functional IDRs showing disorder-order transitions upon binding to their interaction partners [39–41]. These regions are called protean segments (ProSs) [37, 38]. We will introduce an example, as follows (see other two cases in S1 Document).

Among the Smad family proteins that play a pivotal role in the TGF-β signaling pathway, Smad3 (mothers against decapentaplegic homolog 3, IDEAL identifier: IID00113) shows the interesting features of IDRs involved in both translocation from the cytoplasm to the nucleus and PTM. Smad3 consists of two structural domains, called MH1 and MH2. These two domains are connected by a linker region, which is disordered in the unbound state [42]. Another short IDR flanking the C-terminus of the MH2 domain has phosphorylation sites, which are phosphorylated by the receptor kinase, TGF-β [42]. When the C-terminal IDR is phosphorylated, Smad3 forms a trimer with another Smad3 and a Smad4 (IID00132), to translocate into the nucleus. Since Smad3 is a transcription factor, it induces the expression of many genes regulated by TGF-β. The linker IDR also has phosphorylation sites, and the phospho-serines in this region are recognized by the WW domains of a ubiquitin-ligase, NEDD4 (IID00114) [43]. The poly-ubiquitination of the linker IDR, by NEDD4, causes the proteasome system to degrade Smad3 to suppress its transcriptional activity. While the linker IDR is disordered in the isolated state, it becomes structured upon binding to NEDD4. This linker IDR is a typical protean segment (ProS). Smad 3 has several ProSs, including the phosphorylation sites in the linker and C-terminal regions. More details are available on the entry page of IID00113.

#### CMP related to post-translational modifications or nucleotide-binding, and NCMP

Tables 2 and 3 indicate all hub proteins of CMP and NCMP, respectively, in the descending order of the number of interaction partners. All proteins in Table 2 are also annotated with PTM’ + nucleotide-binding keywords. The functions in both Tables are generally similar, in that they are involved in the signaling pathways and interact with membrane receptors and other proteins.

**Table 2:** Hub proteins localized in both the cytoplasm and cell membrane, annotated by PTM’ or nucleotide-binding.

protein	Uniprot	HPRD	PPI	keyword	length	%ID	LID	TM	domains	function	process	step	IDEAL
Tyrosine-protein kinase Lck	P06239	1080	105	Ac/Nb/U	509	13	64	0	3	PK	T-Cell	2	
E3 ubiquitin-protein ligase CBL	P22681	1320	85	U	906	53	426	0	5	UbL	Tyr-K	2	IID00300
Tyrosine-protein kinase SYK	P43405	2514	74	Nb/U	635	17	100	0	2	PK	B-cell	2	
E3 ubiquitin-protein ligase SMURF1	Q9HCE7	6902	74	U	757	21	85	0	3	UbL	BMP	1	IID00328
Adapter molecule crk	P46108	1267	65	Ac	304	4	13	0	3	AP	Reeling	2	
Mast/stem cell growth factor receptor Kit	P10721	1287	54	Nb/U	976	11	41	46	5	RC	VA	0	
Tyrosine-protein kinase ZAP-70	P43403	1495	48	Ac/Nb	619	2	13	0	2	PK	T-Cell	1	
Guanine nucleotide-binding protein G(i) subunit α-2	P04899	764	48	Nb	355	2	5	0	1	MD	VA	1	

Abbreviations: Uniprot, Uniprot accession; HPRD, HPRD ID; PPI, number of PPIs; in the keyword column, Ac, acetylation; Nb, nucleotide-binding; U, Ubl conjugation; %ID, percentage of IDR; LID, length of the longest IDR; TM, length of the predicted trans-membrane regions; domains, number of domains; in the function column, PK, protein kinase; UbL, ubiquitin ligase; AP, adaptor in signaling pathways; RC, receptor; MD, Modulator in signaling pathways; in the process section, T-Cell, T-Cell signaling pathway; Tyr-K Tyrosine kinase signaling pathway; B-Cell, B-cell signaling pathway; BMP, BMP signaling pathway; Reeling, Reeling signaling pathway; VA, various signaling processes; step, number of steps from the cell membrane in KEGG pathway maps; IDEAL, IDEAL identifier.

**Table 3:** Hub proteins localized in the nucleus, cytoplasm and cell membrane.

protein	Uniprot	HPRD	PPI	keyword	length	%ID	LID	TM	domains	function	process	step	IDEAL
Tyrosine-protein kinase Fyn	P06241	655	154	P/Nb	537	16	86	0	3	PK	sphingolopid	1	
RAC-α serine/threonine-protein kinase (Akt1)	P31749	1261	117	P/Ac/U/Nb	480	0	3	0	3	PK	HIF-1	2	IID00412
Glycogen synthase kinase-3 β	P49841	5418	73	P/Nb	420	16	35	0	1	PK	Wnt	2	IID00052
Protein NDRG1	Q92597	5586	61	P/Ac	394	21	83	0	1	RP	stress		
Tyrosine-protein kinase BTK	Q06187	2248	56	P/Ac/T/Nb	659	6	40	0	5	PK	NF-κΒ	2	
Guanine nucleotide-binding protein G(i) subunit α -1	P63096	756	50		354	3	7	0	1	MD	cGMP	1	
Receptor tyrosine-protein kinase erbΒ-2	P04626	1281	46	P/T/Av	1255	28	262	46	3	R-PK	ErbΒ	0	IID00293
Protein kinase C ε type	Q02156	1500	45	P/Nb	737	4	31	0	4	PK	sphingolopid	3	IID00066
Peripheral plasma membrane protein CASK	O14936	2164	37	P/Nb	926	14	43	0	5	SC	adhension		
Catenin δ-1	O60716	3026	22	P/Ac/T	968	48	357	0	1	RB	Wnt	1	

Abbreviations: Uniprot, Uniprot accession; HPRD, HPRD ID; PPI, number of PPIs; in the keyword column, P, phosphoprotein; Ac, acetylation; U, Ubl conjugation; T, transcription; Nb, nucleotide-binding; %ID, percentage of IDR; LID, length of the longest IDR; TM, length of the predicted trans-membrane regions; domains, number of domains; in the function column, PK, protein kinase; RP, response protein; MD, Modulator in signaling pathways; R-PK, receptor type protein kinase; SC, scaffold protein; RB, receptor binding; in the process section, sphingolipid, sphingolipid signaling pathway; HIF-1, HIF-1 signaling pathway; Wnt, Wnt signaling pathway; stress, stress response; NF-κΒ, NF-κΒ signaling pathway; cGMP, cGMP-PKG signaling pathway; ErbΒ, ErbΒ signaling pathway; adhesion, cell adhesion; step, number of steps from the cell membrane in KEGG pathway maps; IDEAL, IDEAL identifier.

Typical examples of CMP are the Src family kinases, Lck and SYK. They are non-receptor type tyrosine kinases composed of the kinase, SH2 and SH3 domains, together with a membrane-targeting region at the N-terminus. They associate with plasma membrane and interact with growth factor receptors to regulate cell growth and proliferation [44, 45]. Referring to the functional descriptions in Uniprot [25], most of the proteins in Table 2 associate with membrane receptors. Table 2 also contains two ubiquitin ligases, CBL and SMURF1. In addition to its presence in two subcellular compartments, SUMRF1 is reportedly localized in the nucleus, where it forms a complex with Smad7 to regulate TGF-β signaling [46]. Although it is unclear whether these ligases are NCMP, the SMURF1-Smad7 complex interacts with the membrane-bound TGF receptor, and functions in the initial steps of the signaling pathway. In the “step” column, we show the number of steps in KEGG [47] from the membrane receptors to the corresponding proteins in the signaling pathways. In general, the number of steps is small, indicating that the hub proteins in CMP with PTM’ + nucleotide-binding keywords can be upstream proteins in the signaling pathways.

The hub proteins of NCMP in Table 3 also include several kinases. RAC-α serine/threonine-protein kinase (Akt1) belongs to the AKT kinase family, which regulates many biological processes including metabolism, proliferation, and cell survival. About 10% of cytosolic Akt1 reportedly exists in lipid raft membranes [48], βand it shuttles between the cytoplasm and the nucleus by co-localizing with the serine-threonine kinase Mst1 [49]. Akt1 was found only in the cytoplasm when it was transfected alone, whereas it was found in the nucleus when co-transfected with Tcl1 [50].

In summary, the hub proteins of CMP and NCMP can be the initiators of signaling pathways to associate with membrane-bound receptors, and they translocate into the cytoplasm (and the nucleus) by interacting with many partner proteins.

### Multiple post-translational modifications and multiple-localization on hub proteins

**Fig 5:**
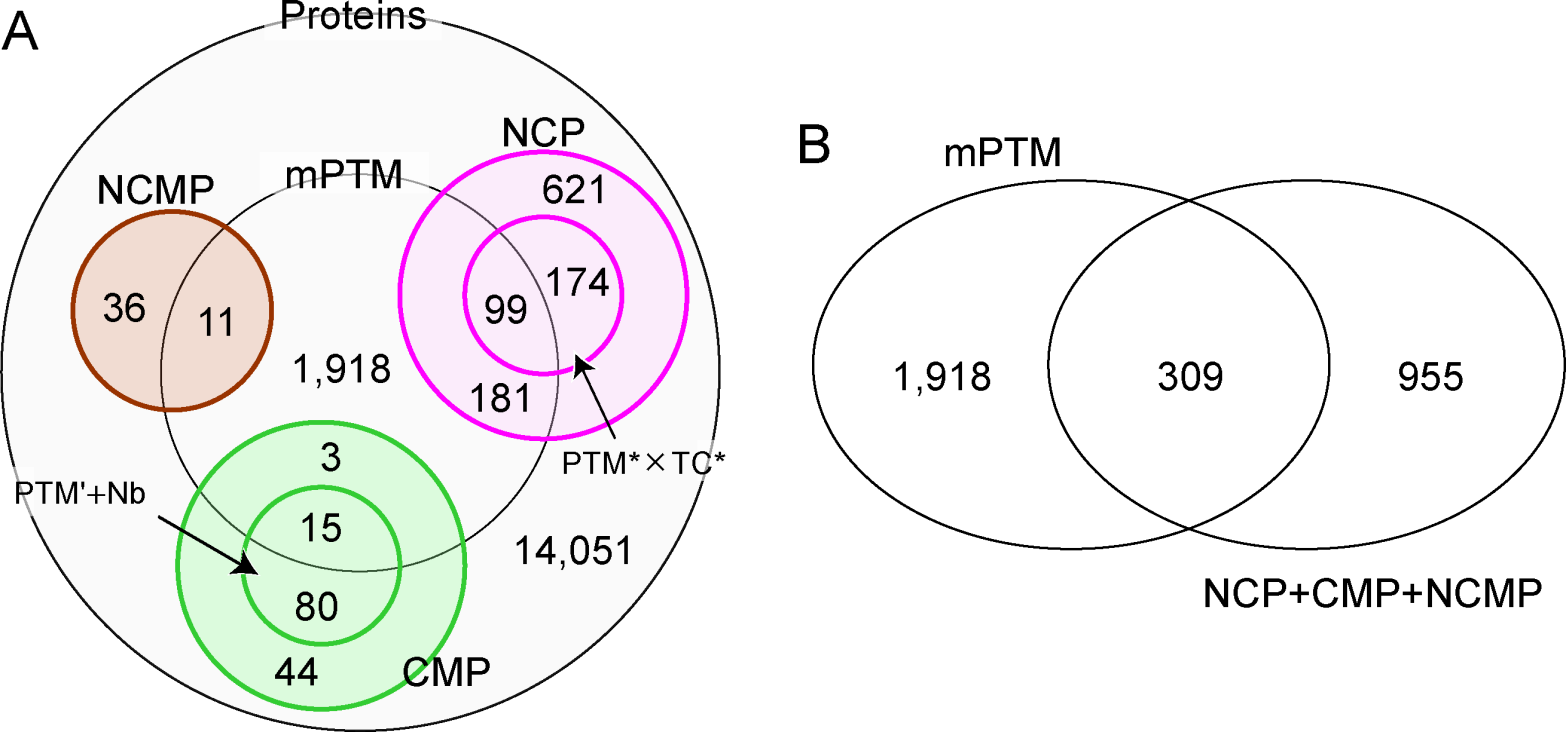
Venn diagrams showing the overlap of the proteins that undergo post-translational modifications multiple times (mPTM) [27], and the multiple-localized hub proteins. The mPTM proteins were obtained from Supplementary File 2 of [27]. We regarded mPTM proteins as proteins that undergo PTM more than once (exactly speaking, the classification group symbol in the File is at least 2), and did not decompose further classifications. Proteins with PTM that were not annotated in the File were disregarded. A. Detailed classification. The small circles in NCP and CMP are the proteins with PTM* × Transcription* (TC*) and those with PTM’ + Nucleotide-binding (Nb), respectively. B. Schematic classification.

Huang et al. investigated the proteins that undergo post-translational modifications multiple times (mPTM), and reported their strong correlation with hub-proteins as well as disease proteins [27]. They also pointed out that such proteins tend to be localized in the nucleus/cytoplasm. Then, the question arises as to whether the mPTM proteins are (almost) the same as the multiple-localized hub proteins discussed here. We examined the relationship between the mPTM proteins and NCP, CMP and NCMP, referring to the original data of Huang et al. [27]. The inclusive relationship is shown in Fig 5. Among 17,233 proteins analyzed using Huang’s data and ours, 2,227 are mPTM proteins (the center circle in Fig 5A), and 15,006 are not (the large circle minus the center circle). Among the multiple-localized hub proteins, 309 are included in mPTM proteins, but 955 are not (Fig 5B). Among the 1,264 multiple-localized hub proteins, less than a quarter overlapped with the mPTM proteins. The Jaccard index is quite small, and less than 0.1 (309/(2,227+955)). Consequently, we conclude that the apparent inclusion relation or overlap was not recognized, and it would be difficult to consider the two groups are identical or strongly related in nature. Note that it does not conflict with the statement in [27]: it described that mPTM proteins contained abundant NCP, but it does not necessarily mean that mPTM proteins and the multiple-localized hub proteins are almost the same.

## Conclusions

We demonstrated that NCP, CMP and NCMP, tend to be hub proteins. The same results were qualitatively obtained using another dataset [2] (S10 Table), and thus this finding is robust. NCP involved in post-translational modifications and transcription participate in numerous interactions. Actually, the typical hub proteins in this group are transcription factors or regulators in several signaling pathways. They translocate from the cytoplasm to the nucleus, in a manner regulated by phosphorylation and/or ubiquitination. The representative hubs of CMP and NCMP, are kinases or ubiquitin ligases on the cytoplasmic side of the cell membrane that act as upstream regulators of the signaling pathways. To respond to signals from the outside of the cell, and to mediate gene expression conducted in the nucleus, the multiple-localized hub proteins transfer biological information through the cell membrane and the cytoplasm, accompanied by their own translocation in a manner regulated by phosphorylation and/or ubiquitination. In terms of interactions, the multiple-localized hub proteins behave as if they are the local proteins that only function in that particular subcellular compartment (e.g., NP or CP); that is, the interaction partners are shared. Since this manner of multiplicity is generally responsible for the increased number of interaction partners, multiple-localization or translocation represents a universal concept for the hubs in any interaction network.

## Materials and Methods

### Datasets

The PPI data were obtained from HPRD [24], which contained 39,240 interactions for 19,651 proteins. Referring to the Uniprot accession, each entry in HPRD was assigned to an entry in Uniprot [25]. When a single Uniprot entry was assigned to different entries in HPRD, the HPRD entries were joined. The unions of the interaction partners for the HPRD entries were considered as the interaction partners for the unified entry. The redundancy of the PPI data was removed, and the homo-oligomeric interactions were discarded. As a result, we compiled 36,939 interactions for 19,461 proteins.

### Subcellular localization

In Uniprot [25], the subcellular localizations of proteins are described in a hierarchical manner on the lines beginning with the “CC -!- SUBCELLULAR LOCATION” tag (see http://www.uniprot.org/help/subcellular_location in detail). Each of subcellular compartments is terminated by “.”, and thus the multiple-localization, for instance NCP, is shown as “Nucleus. Cytoplasm.”. Some subcellular compartments contain multiple words, divided by “,”, e.g., “Cytoplasm, cytoskeleton.” and “Nucleus, nucleolus.”. In such cases, we only considered the first term, that is, “Cytoplasm” for the former, and “Nucleus” for the latter. Note that the first term always includes the second one, as Uniprot adopts a hierarchical description.

### Keywords

The keywords in Uniprot [25] were analyzed. We identified about 700 kinds of keywords denoted for the HPRD entries. We disregarded “reference proteome”, “complete proteome”, “3D-structure”, and “direct protein sequencing”, as trivial keywords. “Transcription regulation” and “Ubl conjugation pathway” were ignored, because they are almost the same as “transcription” and “Ubl conjugation”, respectively, and thus redundantly assigned to a Uniprot entry. In the analysis of CMP and NCMP, “transferase” was ignored, because it overlapped with “kinase”. The keywords regarding the localization; e.g., “nucleus”, “cytoplasm” and “cell membrane”, were also disregarded because they apparently overlapped with the annotations of the aforementioned subcellular localizations.

### Statistical test

The Mann-Whitney U test was applied to estimate the difference of distributions. P-values were obtained using R, and are shown in S2–S5 and S8 Tables. For estimation of keywords enrichment, Z-scores were derived based on the binomial and the normal distributions (see S1 Document and S7 Table for details).

### Hub proteins

We focused on sets of proteins, calculated the average numbers of interactions, and compared their relative values. The distributions of the number of interactions were evaluated statistically, and those with significantly large numbers of interactions were identified. For instance in S3 Table, NCP show a highly statistically significant difference (the p-value: 4.9 × 10^−14^) with all bi-localized proteins. The hub proteins are defined as the ones that are included in such sets and contribute to numerous interactions. Thus, if enough hub proteins are subtracted from the sets, the statistical significances (see S3 and S4 Tables) disappear. Using this definition, we determined the threshold numbers of interactions for hub proteins above which the p-value becomes larger than 0.01 by the subtraction. The hub proteins in NCP, CMP, and NCMP were thereby defined to be the proteins that interact with more than or equal to 14, 48 and 22 proteins, respectively. Tables 2 and 3 list all the hub proteins of CMP and NCMP. The complete list of hub proteins of NCP is shown in S9 Table.

## Supporting Information

S1 Document Method to evaluate the enrichment of keywords. The relation between the number of proteins with a keyword, and the effect of elimination of the proteins on the average number of interactions. Breakdown of multi-domain proteins. Additional examples of NCP related to post-translational modifications and transcription. Cross talk in the signaling pathways.
(PDF)

S1 Fig The average number of interactions against the number of subcellular compartments (Full size view of Fig 1C). (DOCX)

S2 Fig The average number of interactions decomposed by the interaction partners in 7 categories (Full size view of Fig 2A). (DOCX)

S3 Fig The distributions of protein length and longest IDR length (Full size view of Fig 4). (DOCX)

S1 Table Average number of protein interactions in each of the subcellular localizations. (XLSX)

S2 Table P-values of Mann-Whitney U test for the number of interactions: effect of an increment in the number of subcellular compartments. (DOCX)

S3 Table P-values of Mann-Whitney U test for the number of interactions: effect of two specific subcellular compartments. (DOCX)

S4 Table P-values of Mann-Whitney U test for the number of interactions: effect of three specific subcellular compartments. (DOCX)

S5 Table P-values of Mann-Whitney U test for the number of interactions: effect of four specific subcellular compartments. (DOCX)

S6 Table Share rates of interaction partners. (DOCX)

S7 Table Z-scores of enriched keywords. (DOCX)

S8 Table P-values of Mann-Whitney U test for the protein structural features against those of all proteins. (DOCX)

S9 Table Hub proteins localized in both the nucleus and cytoplasm. (XLSX)

S10 Table Average number of interactions derived using the ComPPI data. (DOCX)
