# Supplementary material for: Multiple-Localization and Hub Proteins
Source: PLoS One. 2016 Jun 10;11(6):e0156455. doi: 10.1371/journal.pone.0156455 (PMC4902230; doi:10.1371/journal.pone.0156455)
Supplement: S1 Document — The relation between the number of proteins with a keyword, and the effect of elimination of the proteins on the average number of interactions. Breakdown of multi-domain proteins. Additional examples of NCP related to post-translational modifications and transcription. Cross talk in the signaling pathways. (PDF) [file pone.0156455.s001.pdf]

Supporting Document 1

Multiple-localization and hub proteins

Motonori Ota [1,*], Hideki Gonja [1], Ryotaro Koike [1] and Satoshi Fukuchi [2]
1. Nagoya University, 2. Maebashi Institute of Technology, * Corresponding author

Enrichment of keywords.

To characterize the specific functions and features of NCP, CMP and NCMP, we analyzed the Uniprot keywords [1]. The enrichments of each keyword in NCP, CMP and NCMP as well as NP, CP and MP were evaluated, using the Z score (S7 Table). From the ensemble of all keywords denoted in all HPRD entries [2], we derived the probability of the appearance for each keyword (p). When we found n entries with a given keyword in an ensemble of N entries, the enrichment was estimated by the binomial distribution B(N, p) [3], and the distribution was approximated by the normal distribution N(Np, Np(1-p)). The Z score was defined by (n-av)/div, where av=Np and div=$\sqrt{Np(1-p)}$. We only assessed keywords that appeared more than 500 times in total (55 keywords).

The relation between the number of proteins with a keyword, and the effect of elimination of the proteins on the average number of interactions.

To identify the critical features characterizing NCP, CMP and NCMP, we focused on a keyword, and calculated the decrease in the average number of interactions if the proteins with the keyword were eliminated from the statistics. The plot of the rate of decrease and the frequency of the number of the eliminated proteins (Fig. 3) revealed that most of the data were concentrated in the upper left region, and those in the bottom right region were sparse. The dashed gray line was drawn to show the border of the regions (the origin and the phosphoprotein data was connected). We tried to interpret the line. When we eliminated the proteins with the keyword j, the decrease Dj is written as, $D_j = \frac{I}{N} - \frac{I - i_j}{N - n_j}$, where I, N, i_j and n_j are the total number of interactions, the total

number of proteins, the total number of interactions of proteins with the keyword j, and the total number of proteins with the keyword j, respectively. This equation is transformed to $D_j = (\alpha_j - \alpha)\frac{x_j}{1 - x_j}$, where $\alpha_j = i_j/n_j$, $\alpha = I/N$ and $x_j = n_j/N$. If we expand it by x_j, and neglect the higher order terms, we obtain $D_j = (\alpha_j - \alpha)x_j$. In the actual case, the x_j values are not small, but we confirmed that the slope of the dashed gray line in Fig. 3 is related to $1/(\alpha_j-\alpha)$, and the line works as a separator of α_j. The data above the dashed gray line represent the proteins with α_j values smaller (α_j of alternative splicing: 10.7) than those of the proteins on the dashed gray line; for example, the proteins with activator, transcription, or acetylation, which have α_j values of 16.6, 16.7 and 15.0, respectively. If α_j is large (Ubl conjugation: 24.0), then the data are plotted below the dashed gray line.

Breakdown of multi-domain proteins.

We noticed that the average percentages of multi-domain proteins were similar in the NP (69.7%) and NCP annotated by PTM* × transcription* keywords (69.2%), but the contents were different. We divided the multi-domain proteins into three types: proteins composed of only distinctive domains (D), only repetitive domains (R), and both distinctive and repetitive domains (B) [4, 5] (Fig. 4). In NP, the percentage of repetitive domains (R+B) is high (30.0%), but the rate for NCP annotated by PTM* × transcription* is only 12.0%, which is the minimum among all (13.6% (CP), 14.3% (NCP), 16.5% (all)). However, among the latter proteins, the percentage of only distinctive domains (D) is high (57.3%), as compared to that of the former proteins (39.7%). The rates of D+B are similar (66.0% and 66.7%, respectively) in the former and the latter proteins. In NCP related to PTM and transcription, we recognized the strong preference for the multi-distinctive domains, instead of the multi-repetitive domains.

Additional examples of NCP related to post-translational modifications and transcription.

Transcription factor p65 (IDEAL identifier [6,7]: IID00207) belongs to the Rel/NF-kB protein family, and is involved in the NF-κB signaling pathway. The N-terminal region of the protein has a Rel homolog domain (RHD), consisting of a DNA binding region

and a dimerization region. The C-terminus of the RHD domain has a nuclear localization signal (NLS). IDEAL suggested that the region including the NLS is a ProS. The region is disordered in the mouse homolog [8]. Upon the interaction with NF-κB inhibitor alpha (IκB, Uniprot accession: P25963), it folds and becomes ordered. In the p65/IκB complex, the inhibitor covers the NLS and suppresses the translocation activity of p65. When the IκB kinase is activated and phosphorylates IκB, the IκB becomes poly-ubiquitinated for degradation. This degradation of IκB induces the dissociation of the p65/IκB complex and the exposure of the NLS to importin, thus triggering the translocation into the nucleus. Interestingly, this ProS provides a binding site for multiple proteins, such as N-lysine methyltransferase SETD6 (Q8TBK2) and bromodomain-containing protein 4 (IID00111), in addition to IκB. As shown in this example, the promiscuous binding of IDRs facilitates the functions of hub proteins. Furthermore, we noticed that the interactions of the proteins listed in Table 1 included numerous examples of cross-talk between distinct signaling pathways (see the next section).

CREB-binding protein (CBP, IID00092) and p300 (IID00070) are transcriptional co-activators with histone acetyltransferase activity. They are long proteins composed of more than 2,000 residues. The proteins share high sequence similarity (60%) and have 7 distinct structural domains linked with predicted long IDRs. As shown on the entry pages of IDEAL (see also IID50008 of mouse CBP), these domains have many different binding partners involved in transcription processes. In transcription, numerous proteins gather, interact to construct the transcription machinery and then dissociate. Accordingly, the main functional role of the IDRs in CBP/p300 is suggested to be flexible linkers tethering the structural domains, so that the multiple binding partners are localized and interact with high probability. Hence, CBP and p300 act as transcriptional scaffolds [9].

Cross-talk in the signaling pathways.

We noticed that some of the hub proteins in Table 1 interact with each other [2], although they are involved in distinct signaling pathways. For example, p65 in the NF-κB signaling pathway interacts with STAT3 in the JAK/STAT signaling pathway, and smad3 in the smad signaling pathway interacts with androgen receptor in the nuclear receptor signaling pathway. In addition, the former interacts with STAT1 and 6 (IID00046 and 47, JAK/STAT signaling pathway) and notch1 (IID00199, notch

signaling pathway), and the latter interacts with axin-1 (IID00007, wnt signaling pathway), β-catenine (IID00039, wnt signaling pathway) and notch1. These interaction partners, which are not listed in Table 1, also translocate from the cytoplasm to the nucleus. As shown in these instances, the numerous intra-interactions within NCP represent the cross-talk among signaling pathways, suggesting that the pathway cross-talk contributes to the enrichment of the intra-interactions of NCP (Fig. 2).

References

1. UniProt-Consortium. Ongoing and future developments at the Universal Protein Resource. Nucleic Acids Res. 2011; 39 (Database issue): D214-219.
2. Keshava Prasad TS, Goel R, Kandasamy K, Keerthikumar S, Kumar S, Mathivanan S, et al. Human Protein Reference Database--2009 update. Nucleic Acids Res. 2009; 37 (Database issue): D767-772.
3. Koike R, Ota M, Kidera A. Hierarchical description and extensive classification of protein structural changes by Motion Tree. J Mol Biol. 2014; 426 (3): 752-762.
4. Patil A, Kinoshita K, Nakamura H. Domain distribution and intrinsic disorder in hubs in the human protein-protein interaction network. Protein Sci. 2010; 19 (8): 1461-1468.
5. Ekman D, Light S, Bjorklund AK, Elofsson A. What properties characterize the hub proteins of the protein-protein interaction network of Saccharomyces cerevisiae? Genome Biol. 2006; 7 (6): R45.
6. Fukuchi S, Sakamoto S, Nobe Y, Murakami SD, Amemiya T, Hosoda K, et al. IDEAL: Intrinsically Disordered proteins with Extensive Annotations and Literature. Nucleic Acids Res. 2012; 40 (Database issue): D507-511.
7. Fukuchi S, Amemiya T, Sakamoto S, Nobe Y, Hosoda K, Kado Y, et al. IDEAL in 2014 illustrates interaction networks composed of intrinsically disordered proteins and their binding partners. Nucleic Acids Res. 2014; 42 (Database issue): D320-325.
8. Huang DB, Huxford T, Chen YQ, Ghosh G. The role of DNA in the mechanism of NFkappaB dimer formation: crystal structures of the dimerization domains of the p50 and p65 subunits. Structure. 1997; 5 (11): 1427-1436.
9. Dyson HJ, Wright PE. Intrinsically unstructured proteins and their functions. Nat Rev Mol Cell Biol. 2005; 6 (3): 197-208.
