# Supplementary material for: Multiple-Localization and Hub Proteins
Source: PLoS One. 2016 Jun 10;11(6):e0156455. doi: 10.1371/journal.pone.0156455 (PMC4902230; doi:10.1371/journal.pone.0156455)
Supplement: S1 Fig — (DOCX) [file pone.0156455.s002.docx]

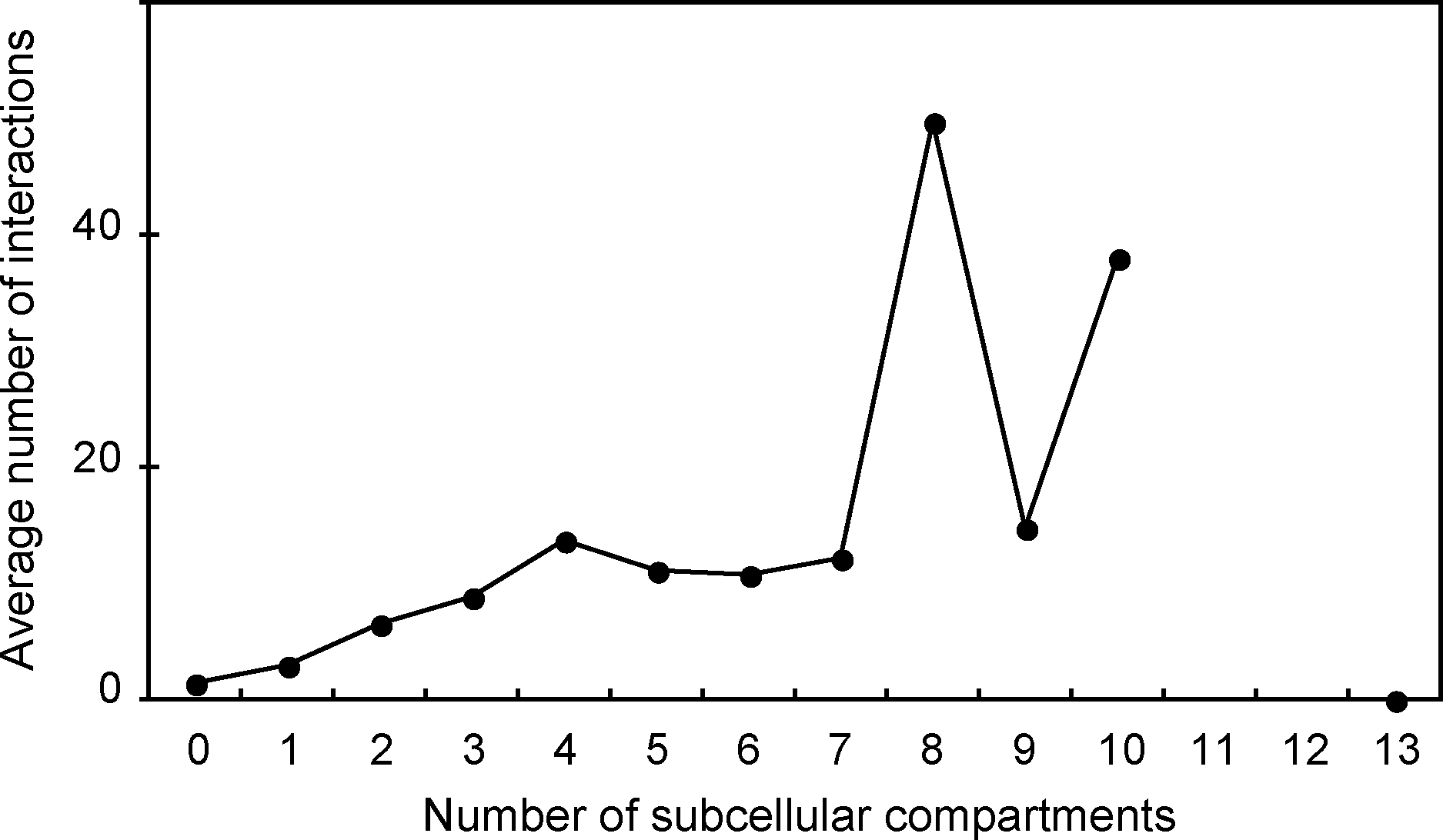


S1 Figure. The average number of interactions against the number of subcellular compartments (Full size view of Fig. 1C).
