# Supplementary material for: Multiple-Localization and Hub Proteins
Source: PLoS One. 2016 Jun 10;11(6):e0156455. doi: 10.1371/journal.pone.0156455 (PMC4902230; doi:10.1371/journal.pone.0156455)
Supplement: S2 Fig — (DOCX) [file pone.0156455.s003.docx]

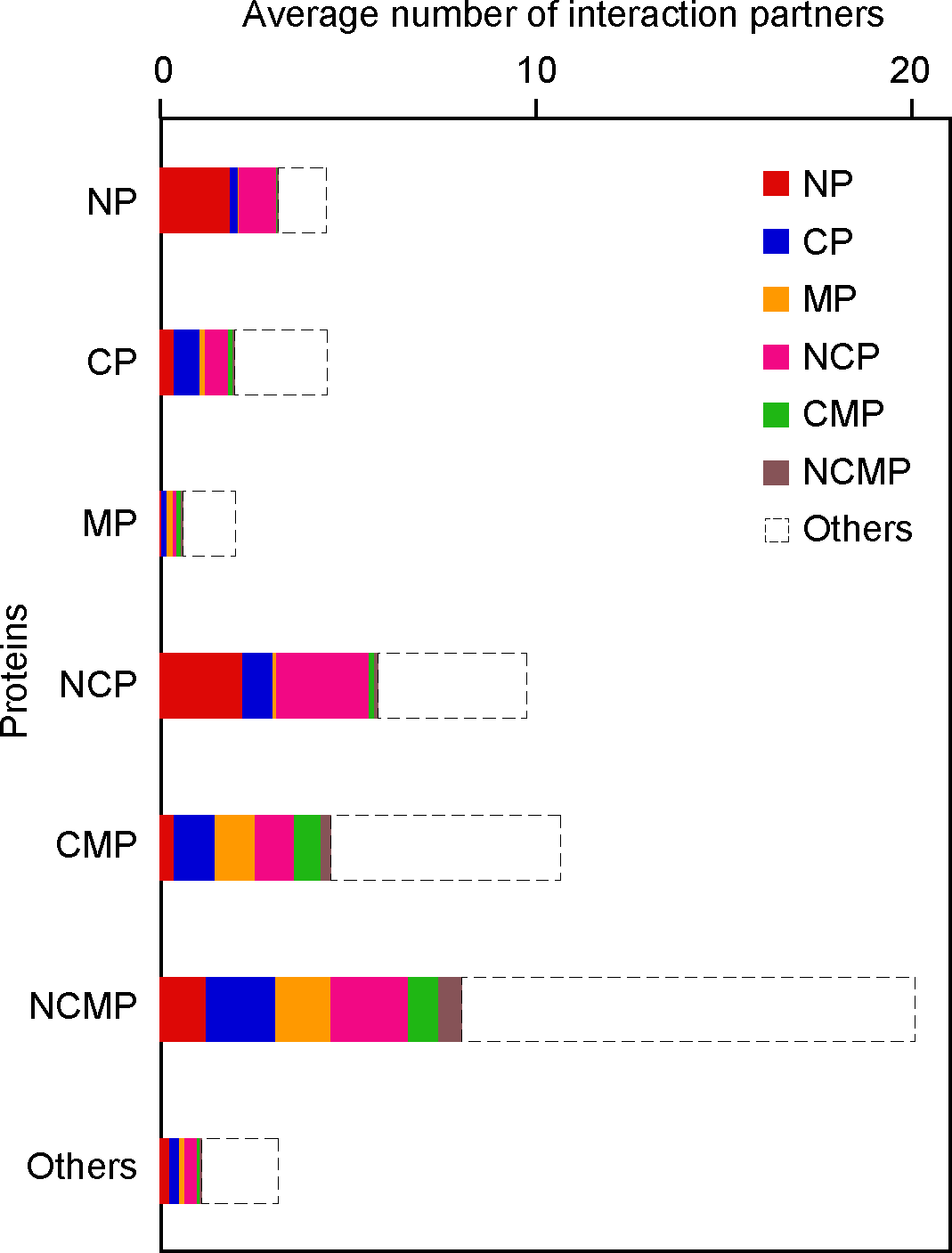


S2 Figure. The average number of interactions decomposed by the interaction partners in 7 categories (Full size view of Fig. 2A).
