# Supplementary material for: Multiple-Localization and Hub Proteins
Source: PLoS One. 2016 Jun 10;11(6):e0156455. doi: 10.1371/journal.pone.0156455 (PMC4902230; doi:10.1371/journal.pone.0156455)
Supplement: S3 Fig — (DOCX) [file pone.0156455.s004.docx]

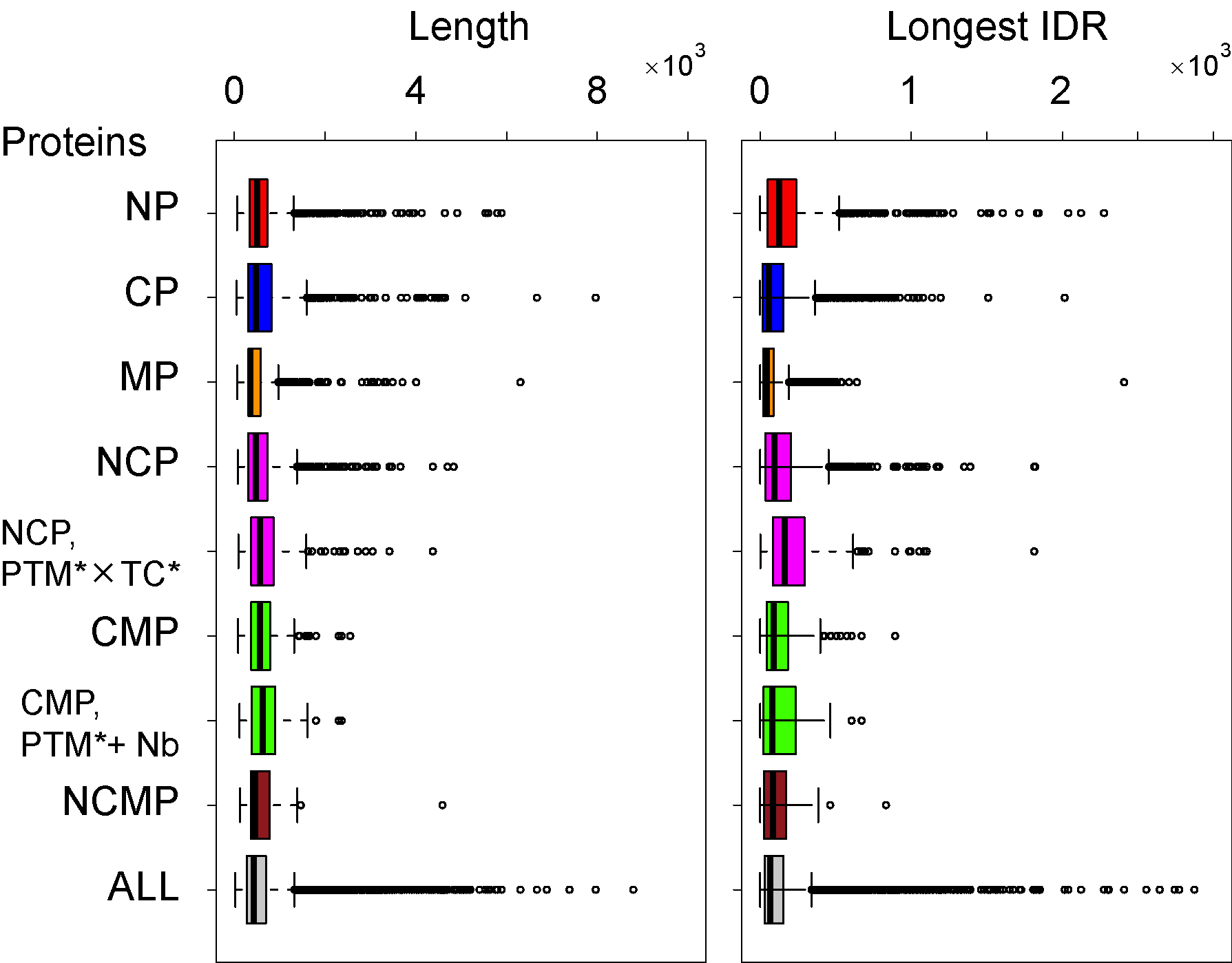


S3 Figure. The distributions of protein length and longest IDR length (Full size view of Fig. 4). A few numbers of large outliers are ignored.
