# Supplementary material for: Multiple-Localization and Hub Proteins
Source: PLoS One. 2016 Jun 10;11(6):e0156455. doi: 10.1371/journal.pone.0156455 (PMC4902230; doi:10.1371/journal.pone.0156455)
Supplement: S2 Table — (DOCX) [file pone.0156455.s006.docx]

Table S2: P-values of Mann-Whitney U test for the number of interactions:

effect of an increment in the number of subcellular compartments

Set1 Proteins in Set1 Set2 P-value

All2 3,377 All1 *

All3 955 All2 1.3 × 10^-6^

All4 323 All3 4.1 × 10^-3^

All5 133 All4 0.83

All6 52 All5 0.30

All7 23 All6 0.52

All8 4 All7 0.11

All9 4 All8 0.17

All10 1 All9 0.80

The distribution of the number of interactions in Set1 and that of Set2 were compared. The number after “All” indicates the number of subcellular compartments; e.g., All1 is the set of all proteins that are localized in only one subcellular compartment, while All2 is the set of all bi-localized proteins. An asterisk (*) indicates a p-value less than 2.2 × 10^-16^. The small p-value means that the distributions are statistically different.
