# Supplementary material for: Multiple-Localization and Hub Proteins
Source: PLoS One. 2016 Jun 10;11(6):e0156455. doi: 10.1371/journal.pone.0156455 (PMC4902230; doi:10.1371/journal.pone.0156455)
Supplement: S3 Table — (DOCX) [file pone.0156455.s007.docx]

Table S3: P-values of Mann-Whitney U test for the number of interactions:

effect of two specific subcellular compartments

Subcellular localizations Number of proteins P-value

Nucleus/Cytoplasm (NC) 1,120 4.9 × 10^-14^

Cytoplasm/Cell membrane (CM) 146 5.5 × 10^-4^

Cytoplasm/Cell junction 46 0.52

Nucleus/Nucleus speckle 44 0.36

Cell membrane/Membrane 30 0.19

Cytoplasm/ER 15 0.33

Nucleus/Cell membrane 14 0.05

Cytoplasm/Early endosome membrane 11 0.15

The numbers of interactions of proteins localized in two specific subcellular compartments were compared with that of all bi-localized proteins (All2 in Table S2). Sets of proteins with average numbers of interactions greater than the average of All2 (6.51) were examined.
