# Supplementary material for: Multiple-Localization and Hub Proteins
Source: PLoS One. 2016 Jun 10;11(6):e0156455. doi: 10.1371/journal.pone.0156455 (PMC4902230; doi:10.1371/journal.pone.0156455)
Supplement: S4 Table — (DOCX) [file pone.0156455.s008.docx]

Table S4: P-values of Mann-Whitney U test for the number of interactions:

effect of three specific subcellular compartments

Subcellular localizations Number of proteins P-value

Nucleus/Cytoplasm/Cell membrane (NCM) 49 1.6 × 10^-5^

Nucleus/Cytoplasm/Membrane 41 0.80

Nucleus/Cytoplasm/Mitochondrion 35 0.23

Cytoplasm/Cell membrane/Cell junction 19 0.03

Nucleus/Cytoplasm/Cell junction 15 0.20

Nucleus/Cytoplasm/Secreted 14 0.11

Nucleus/Cytoplasm/ER 13 0.25

Cell membrane/Cell junction/Cell projection 12 0.13

The numbers of interactions of proteins localized in the three specific subcellular compartments were compared with that of all tri-localized proteins (All3 in Table S2). Sets of proteins with average numbers of interactions greater than the average of All3 (8.86) were examined.
