# Supplementary material for: Multiple-Localization and Hub Proteins
Source: PLoS One. 2016 Jun 10;11(6):e0156455. doi: 10.1371/journal.pone.0156455 (PMC4902230; doi:10.1371/journal.pone.0156455)
Supplement: S5 Table — (DOCX) [file pone.0156455.s009.docx]

Table S5: P-values of Mann-Whitney U test for the number of interactions:

effect of four specific subcellular compartments

Subcellular localizations Number of proteins P-value

Cytoplasm/Cell membrane/Cell junction/Cell projection 13 0.100

Nucleus/Cytoplasm/Cell membrane/Cell junction 8 0.013

Nucleus/Cytoplasm/Cell membrane/Golgi apparatus 5 0.020

The numbers of interactions of proteins localized in the four specific subcellular compartments were compared with that of all quad-localized proteins (All4 in Table S2). Sets of proteins with average numbers of interactions greater than the average of All4 (13.74) were examined.
