# Supplementary material for: Multiple-Localization and Hub Proteins
Source: PLoS One. 2016 Jun 10;11(6):e0156455. doi: 10.1371/journal.pone.0156455 (PMC4902230; doi:10.1371/journal.pone.0156455)
Supplement: S6 Table — (DOCX) [file pone.0156455.s010.docx]

Table S6: Share rates of interaction partners

Interaction partners

Proteins NP CP MP NCP CMP NCMP

NP - 0.614 0.303 **0.797** 0.476 0.389

CP 0.790 - 0.307 **0.796** 0.517 0.500

MP 0.816 0.633 - 0.839 0.500 0.667

NCP **0.771** **0.559** 0.357 - 0.490 0.480

CMP 0.796 **0.695** 0.436 **0.816** - 0.571

NCMP **0.906 0.727** 0.463 **0.847 0.889** -

When examining proteins A and interaction partners B, the number of shared interaction partners between B interacting with A, and B having intra-interaction was counted. This number was divided by the number of B interacting with A. The bold numbers represent average numbers of (decomposed) interactions greater than 0.6 (see Fig. 2A), with a share rate greater than 0.5 (S symbol in Fig. 2C).
