# Supplementary material for: Multiple-Localization and Hub Proteins
Source: PLoS One. 2016 Jun 10;11(6):e0156455. doi: 10.1371/journal.pone.0156455 (PMC4902230; doi:10.1371/journal.pone.0156455)
Supplement: S7 Table — (DOCX) [file pone.0156455.s011.docx]

Table S7: Z-scores of enriched keywords

Keywords NP CP MP NCP CMP NCMP

Phosphoprotein 11.3 - - 20.8 5.3 6.0

Transmembrane - - 52.7 - - -

Repeat 18.8 - - - - -

Glycoprotein - - 52.0 - - -

Metal-binding 29.4 - - - - -

Acetylation - 12.7 - 16.9 - -

Signal - 13.2 - - -

Disulfide bond - - 44.3 - - -

Transcription 82.4 - - 18.3 - -

Zinc 41.8 - - - - -

Coiled coil - 13.9 - - - -

DNA-binding 77.7 - - 10.8 - -

Nucleotide-binding - 14.5 - - 5.9 9.0

Zinc-finger 48.3 - - - - -

Receptor - - 75.5 - - -

ATP-binding - 12.1 - - - 8.3

Ubl conjugation - - - 17.3 - -

Cytoskeleton - 36.8 - - 10.9 -

Developmental protein 16.4 - - - - -

Transducer - - 87.3 - - -

G protein coupled receptor - - 88.6 - - -

Lipoprotein - - 24.9 - 7.3 7.0

Kinase - - - - 6.2 13.3

Activator 35.2 - - 14.9 - -

RNA-binding 10.4 - - 19.4 - -

Cell cycle - - - 10.0 - -

Repressor 33.7 - - 13.6 - -

Sensory transduction - - 58.8 - - -

Only keywords with a Z-score greater than 10 (~7.6 × 10 ^-24^ p-value) are shown in the descending order of the observations of Uniprot keywords in all HPRD entries used in the study. For CMP (only 146 observations) and NCMP (49), Z-scores more than 5 are indicated. “Transcription regulation” and “Ubl conjugation pathway” are omitted, as they are similar to “transcription” and “Ubl conjugation”, respectively. In CMP and NCMP, “Transferase” is ignored, as it is similar to “Kinase”. Also, apparent keywords for localization, “Nucleus”, “Cytoplasm”, “Membrane” and “Cell membrane”, are discarded.
