# Supplementary material for: Multiple-Localization and Hub Proteins
Source: PLoS One. 2016 Jun 10;11(6):e0156455. doi: 10.1371/journal.pone.0156455 (PMC4902230; doi:10.1371/journal.pone.0156455)
Supplement: S8 Table — (DOCX) [file pone.0156455.s012.docx]

Table S8: P-values of Mann-Whitney U test for the protein structural features against those of all proteins

Proteins Attributes Value P-value

NP Length long *

Percentage of IDR high *

Longest IDR long *

Number of domains many *

CP Length long 4.4 × 10^-9^

Percentage of IDR low *

Longest IDR short 5.8 × 10^-6^

Number of domains 0.087

MP Length 0.017

Percentage of IDR low *

Longest IDR short *

Number of domains few 8.5 × 10^-4^

NCP Length long 7.4 × 10^-4^

Percentage of IDR high 6.9 × 10^-10^

Longest IDR long 9.8 × 10^-12^

Number of domains many 3.2 × 10^-4^

NCP, PTM*×TC* Length long 1.9 × 10^-11^

Percentage of IDR high *

Longest IDR long *

Number of domains many 4.8 × 10^-9^

CMP Length long 3.3 × 10^-5^

Percentage of IDR 0.626

Longest IDR 6.3 × 10^-3^

Number of domains many 4.0 × 10^-5^

CMP, PTM’+Nb Length 3.0 × 10^-3^

Percentage of IDR 0.165

Longest IDR 0.747

Number of domains many 6.3 × 10^-4^

NCMP Length 0.12

Percentage of IDR 0.50

Longest IDR 0.77

Number of domains 0.80

Values are described if the p-values are less than 10^-3^. An asterisk (*) indicates the p-value is less than 2.2 × 10^-16^.
