# Supplementary material for: Multiple-Localization and Hub Proteins
Source: PLoS One. 2016 Jun 10;11(6):e0156455. doi: 10.1371/journal.pone.0156455 (PMC4902230; doi:10.1371/journal.pone.0156455)
Supplement: S10 Table — (DOCX) [file pone.0156455.s014.docx]

Table S10: Average number of interactions derived using the ComPPI data

Localization Number of proteins Average number of interactions

Nucleus 1,687 11.8

Cytosol 1,067 9.5

Membrane 26 0.5

Nucleus/Cytosol 3,231 29.3

Cytosol/Membrane 260 14.8

Nucleus/Cytosol/Membrane 708 33.3

The statistics of human proteins in Swissprot were calculated using the data of interactions and localizations from ComPPI. Note that the interactions with the entries in TrEMBL were discarded.
